# Adrenal hormonal imbalance in acute intermittent porphyria patients: results of a case control study

**DOI:** 10.1186/1750-1172-9-54

**Published:** 2014-04-16

**Authors:** Oscar J Pozo, Josep Marcos, Andreu Fabregat, Rosa Ventura, Gregori Casals, Paula Aguilera, Jordi Segura, Jordi To-Figueras

**Affiliations:** 1 Bioanalysis Research Group, IMIM, Hospital del Mar Medical Research Institute, Doctor Aiguader 88, Barcelona 08003, Spain; 2 Department of Experimental and Health Sciencies, Universitat Pompeu Fabra, Doctor Aiguader 88, Barcelona 08003, Spain; 3 Porphyria Unit, Dermatology Unit, Hospital Clínic, IDIBAPS, University of Barcelona, Villarrroel 170, Barcelona 08036, Spain; 4 Biochemistry and Molecular Genetics Department, Hospital Clínic, IDIBAPS, University of Barcelona, Villarrroel 170, Barcelona 08036, Spain

**Keywords:** Steroids, Urine, Acute intermittent porphyria, Metabolomics

## Abstract

**Background:**

Acute Intermittent Porphyria (AIP) is a rare disease that results from a deficiency of hydroxymethylbilane synthase, the third enzyme of the heme biosynthetic pathway. AIP carriers are at risk of presenting acute life-threatening neurovisceral attacks. The disease induces overproduction of heme precursors in the liver and long-lasting deregulation of metabolic networks. The clinical history of AIP suggests a strong endocrine influence, being neurovisceral attacks more common in women than in men and very rare before puberty. To asses the hypothesis that steroidogenesis may be modified in AIP patients with biochemically active disease, we undertook a comprehensive analysis of the urinary steroid metabolome.

**Methods:**

A case–control study was performed by collecting spot morning urine from 24 AIP patients and 24 healthy controls. Steroids in urine were quantified by liquid chromatography-tandem mass spectrometry. Parent steroids (17-hydroxyprogesterone; deoxycorticosterone; corticoesterone; 11-dehydrocorticosterone; cortisol and cortisone) and a large number of metabolites (N = 55) were investigated. Correlations between the different steroids analyzed and biomarkers of porphyria biochemical status (urinary heme precursors) were also evaluated. The Mann–Whitney U test and Spearman’s correlation with a two tailed test were used for statistical analyses.

**Results:**

Forty-one steroids were found to be decreased in the urine of AIP patients (P < 0.05), the decrease being more significant for steroids with a high degree of hydroxylation. Remarkably, 13 cortisol metabolites presented lower concentrations among AIP patients (P < 0.01) whereas no significant differences were found in the main metabolites of cortisol precursors. Nine cortisol metabolites showed a significant negative correlation with heme precursors (p < 0.05). Ratios between the main metabolites of 17-hydroxyprogesterone and cortisol showed positive correlations with heme-precursors (correlation coefficient > 0.51, P < 0.01).

**Conclusions:**

Comprehensive study of the urinary steroid metabolome showed that AIP patients present an imbalance in adrenal steroidogenesis, affecting the biosynthesis of cortisol and resulting in decreased out-put of cortisol and metabolites. This may result from alterations of central origin and/or may originate in specific decreased enzymatic activity in the adrenal gland. An imbalance in steroidogenesis may be related to the maintenance of an active disease state among AIP patients.

## Background

Acute intermittent porphyria (AIP) is a dominant disorder that results from a partial deficiency of hydroxymethylbilane synthase (HMBS, EC 2.5.1.61) the third enzyme of the heme biosynthetic pathway [1]. Carriers of mutations within the HMBS gene are at risk of presenting acute life-threatening neurovisceral attacks [2]. The incidence of new symptomatic cases has been estimated to be 0.13 per million per year in most European countries [3].

The clinical presentation of AIP includes autonomous, central, motor and sensory symptoms. The patients may present abdominal pain, tachycardia, hypertension and hyponatremia. Neuropathy and muscle weakness can lead to tetraplegia, with respiratory and bulbar paralysis [4,5]. Acute attacks are associated with the induction of 5-aminolevulinate synthase (ALAS-1), the first enzyme of the heme synthesis pathway, resulting in overproduction of heme precursors, aminolevulinic acid (ALA) and porphobilinogen (PBG) in the liver [6]. The exportation of ALA to tissues could be mainly responsible for the neurovisceral symptoms. However, the pathophysiology of the disease and the role of partial heme deficiency in tissues is not completely understood [7]. Acute attacks may be triggered by menstrual hormonal changes, fasting, stress and some therapeutic drugs. Intravenous heme administration is a well established highly effective therapy, albeit with transient effects [2]. Nevertheless, although heme administration may resolve acute crises, in most patients the urinary levels of PBG and ALA usually remain elevated for many years [8]. A few AIP patients develop recurrent acute attacks that may require repeated heme infusions or even liver transplantation for their cure [9].

The clinical history of AIP suggests a strong endocrine influence over disease expression, being neurovisceral attacks more common in women than in men and very rare before puberty or after menopause [7]. Moreover, in some series including few patients, the administration of gonadotropin releasing hormone analogues has been shown to reduce recurrent exacerbations associated with menstruation [10].

AIP patients with biochemically active disease present hepatic involvement with sustained ALAS-1 induction. In these patients, with long-lasting oveproduction of heme-precursors in the liver, we have previously reported low plasma levels of insulin-growth factor 1 [11] and a decrease of 5α-reductase activity in the liver by the calculation of several urinary steroid metabolic ratios [12].

A pilot study by Larion et al. [13] studied circadian rhythms in AIP and found a decrease of plasma cortisol in patients with biochemically active disease. These findings suggested that in addition to hepatic involvement, AIP may be associated with hormonal disturbances originating in the adrenal gland. To test this hypothesis and in order to assess possible abnormalities in steroidogenesis among AIP patients, we undertook a comprehensive urinary target analysis of 70 steroid hormones and metabolites by state-of-the art liquid chromatography–tandem mass spectrometry.

## Methods

### Patients

We studied 24 adult Caucasian Spanish patients with biochemically active AIP (22 women and 2 men, ranging in age from 22 to 54 years, Table 1). All these patients had initially presented an acute porphyria attack, had been diagnosed with AIP and regularly attended thereafter in the Porphyria Unit of the Hospital Clinic of Barcelona for clinical follow-up. AIP was assessed by biochemical and enzymatic analyses according to European Porphyria Initiative recommendations and external quality assessment schemes [14]. Genetic analysis of the HMBS synthase gene confirmed the AIP in all the cases. Some of the HMBS mutations found among these Spanish patients have been previously reported [15].

**Table 1:** Description of the case population studied

	**Gender**	**Age**	**Fertile**	**Heme arginate**	**PBG**	**ALA**	**GFR**
**F1**	F	54	No	No	3	1	>60
**F2**	F	32	Yes	No	7	6	>60
**F3**	F	36	Yes	No	10	6	>60
**F4**	F	37	Yes	No	16	10	>60
**F5**	F	25	Yes	No	16	13	>60
**F6**	F	31	Yes	No	17	15	>60
**M1**	M	52	Yes	No	19	9	>60
**F7**	F	49	Yes	No	23	11	>60
**F8**	F	34	Yes	No	26	21	>60
**F9**	F	35	Yes	No	28	12	>60
**F10**	F	31	Yes	No	29	7	>60
**F11**	F	35	Yes	No	30	23	>60
**F12**	F	34	Yes	No	30	12	>60
**F13**	F	27	Yes	No	32	22	>60
**F14**	F	42	Yes	No	32	21	52.84
**F15**	F	51	No	No	34	31	>60
**F16**	F	22	Yes	No	34	15	>60
**F17**	F	29	Yes	No	35	16	>60
**F18**	F	41	Yes	No	37	27	>60
**F19**	F	31	Yes	No	45	20	>60
**F20**	F	20	Yes	No	52	42	>60
**M2**	M	53	Yes	Yes	57	41	> 60
**F21**	F	31	Yes	Yes	63	40	>60
**F22**	F	41	Yes	Yes	64	40	>60

Heme arginate treatment: Normosang®; 3 mg/Kg; every 2–3 weeks; PBG: porphobilinogen concentrations (nmol/mmol creatinine); ALA: aminolevulinic acid concentrations nmol/mmol creatinine). GFR: glomerular filtration rate (mls/min).

At the initiation of the study and prior to urine collection, the patients did not present symptoms of acute porphyria, but chronic complaints such as altered mood states, fatigue or pain in the back were frequently reported. Exceptionally, 3 patients who presented frequent recurrent attacks were on a prophylactic heme-arginate regime (Normosang®; 3 mg/Kg; every 2–3 weeks). The urine of these patients was collected before the heme-arginate infusions. None of the patients included in the study were treated with luteinizing hormone-releasing hormone (LHRH) agonists.

Independently of the clinical status, all the patients presented increased long-term urinary excretion of the heme precursors PBG and ALA. The concentrations of the heme precursors in the urine samples used for steroid analysis are shown in Table 1.

None of the patients presented other diseases in addition to AIP, and all presented normal liver function. Although in some case-series AIP has been reported to be associated with chronic renal failure [16] which could eventually interfere with the objectives of the study, our series of Spanish AIP patients showed no evidence of an increased incidence of renal disease. All the cases included but one (with a borderline increase of creatinine concentrations in plasma) presented a normal glomerular filtration rate (GFR > 60 mls/min, Table 1) estimated by the MDRD equation [17].

All AIP patients reported following the general dietetic and life-style recommendations for AIP carriers. Prior to urine collection all the patients were specifically interviewed and denied self-prescription or casual intake of porphyrinogenic drugs, synthetic hormones or other substances that could eventually interfere with steroid excretion and therefore bias the study results. Prior to steroid and porphyrin analyses urine integrity and normal pH values were checked in all the samples.

Twenty-four healthy volunteers (22 women and 2 men; age 25–45 years) were recruited from the laboratory staff and included in the study as controls. They all presented normal renal function and were interviewed to discard the consumption of substances that could potentially interfere with steroid metabolism.

All patients and controls were informed of the purpose of the study and written consent was obtained. The research was conducted in accordance with the Declaration of Helsinki Principles and was approved by the Hospital Clinic Ethic Committee. Second morning urine from all patients and controls was obtained between 09.00 h-10.00 h in carefully controlled conditions within the hospital premises. Aliquots were immediately protected from light and frozen at -80°C until analyses.

### PBG and ALA measurements

PBG and ALA were measured by ion-exchange chromatography using the ALA/PBG column test (Bio-Rad GmbH, Munich, Germany). Creatinine and liver enzymes were analyzed by standard methods using ADVIA 2400 equipment (Siemens Medical Solutions Diagnostics, Tarrytown, NY, USA). The concentration of PBG/ALA was normalized to creatinine (mmol/mol of creatinine).

### Urinary steroids

Reference standards for steroid hormones, their metabolites and internal standards were obtained from Steraloids Inc. (Newport, USA), Sigma-Aldrich (St Louis, MO, USA), Merck (Darmstadt, Germany), Toronto Research Chemicals (Toronto, Canada), and NMI (Pymble, Australia) (for more information see reference [18]).

**Figure 1:**
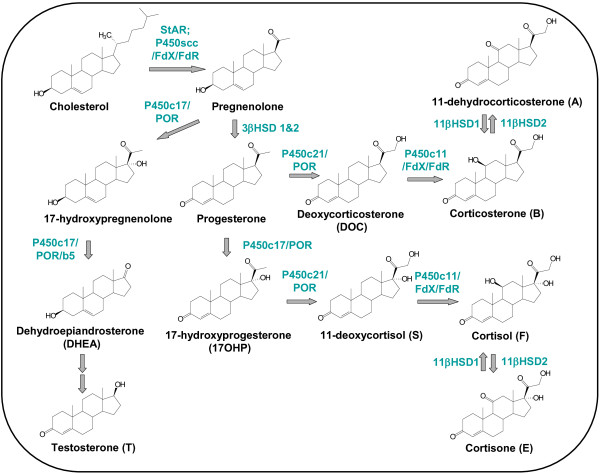
Biosynthesis of steroidal hormones and the enzymes involved.

**Figure 2:**
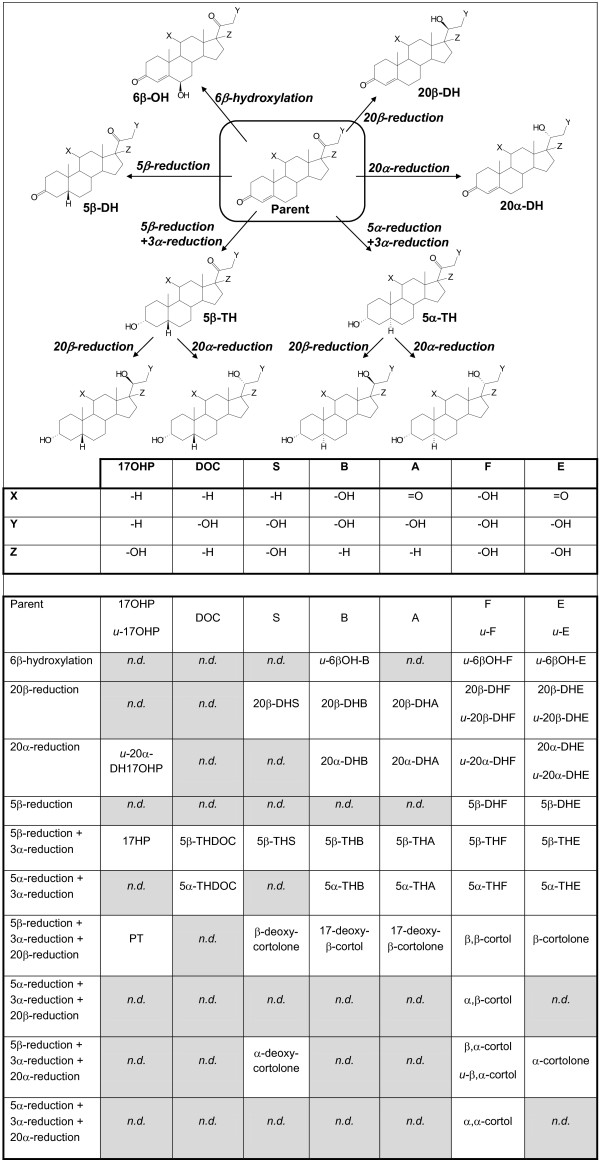
**Schematic representation of C21 steroidal hormone metabolism showing the analytes included in the study.** Of the 70 analytes included in the analytical methods, 55 were detected in a substantial number of samples and therefore included in the evaluation of steroidogenesis in AIP patients. DHEA and T were also included in the study by the analysis of T, androsterone (from 5α-reduction + 3α-reduction of DHEA and from 5α-reduction + 3α-reduction + 17-oxydation of T) and etiocholanolone (from 5β-reduction + 3α-reduction of DHEA and from 5β-reduction + 3α-reduction + 17-oxydation of T) but have been omitted from the figure for clarity.

In summary, the target metabolomic analysis was based on the quantitation of urinary concentrations for 70 analytes including 17-hydroxyprogesterone (17OHP), 11-deoxycortisol (S), deoxycorticosterone (DOC), corticosterone (B), 11-dehydrocorticosterone (A), cortisol (F), cortisone (E) and testosterone (T) and a large number of their metabolites. These hormones represent key steps in steroidogenesis [19] (Figure 1). The structures of the urinary metabolites analyzed are summarized in Figure 2.

### Quantification of urinary steroids

The quantification of urinary steroids was performed by a previously reported method [18]. Briefly, 0.5 mL of urine were added with an internal standard (ISTD) mixture, buffered at pH 7, and enzymatically hydrolysed with β-glucuronidase (60 minutes at 55°C). After hydrolysis, 2 mL of a saturated NaCl solution and 250 μL of a 25% (w/v) K_2_CO_3_ solution were added, and the mixture was extracted with 6 mL of ethylacetate. After evaporation of the organic layer, the extract containing glucuronoconjugated plus unconjugated analytes was reconstituted with 150 μL of water:acetonitrile (9:1, v/v). Unconjugated steroids were also determined by applying the same strategy but omitting the step of enzymatic hydrolysis.

Ten μL of the reconstituted extract were injected into the liquid chromatography-tandem mass spectrometry (LC-MS/MS) system consisting in a triple quadrupole (Xevo) mass spectrometer (Waters Associates, Milford, MA, USA) coupled to an Acquity UPLC system, (Waters Associates) for chromatographic separation. The LC separation was performed using an Acquity BEH C_18_ column (100 mm × 2.1 mm i.d., 1.7 μm) (Waters Associates) at a flow rate of 300 μL min^-1^. Water and methanol both with formic acid (0.01% v/v) and ammonium formate (1 mM) were selected as mobile phase solvents. The detailed gradient and SRM method has been described elsewhere [18].

Quantification was performed after peak area integration of the analytes and the ISTD and comparison with a calibration curve. Results were normalized to creatinine levels (μg/g creatinine).

### Statistical analysis

Among the metabolites determined by the study method, those detected in at least 50% of either the control or cases samples were included in the data analysis. The remaining analytes were discarded.

All the analytes were monitored in both unconjugated and total (conjugated + unconjugated) fractions. The unconjugated data was not considered for analysis among those analytes with a concentration in the unconjugated fraction representing less than 10% of the total amount, In cases in which the unconjugated concentration was greater than 90% of the total, the data obtained in the analysis of the total fraction was discarded from the analysis.

Data were analyzed using the SPSS software (v 18.0; IBM, Armonk, New York, NY, USA). The statistical analysis was used to reveal the differences between the two groups; cases and controls. Correlations between the different compounds analyzed, and the heme precursors (PBG and ALA) were also evaluated. Since the low number of data analyzed hampers the assumption of normality, the statistical analysis was conducted using non-parametric tests. A Mann–Whitney U test was used to compare the differences between the two groups and Spearman’s correlation with a two-tailed test was used for the evaluation of the correlation among all the compounds analyzed. Statistical significance was set at p ≤ 0.05 or p ≤ 0.01.

**Figure 3:**
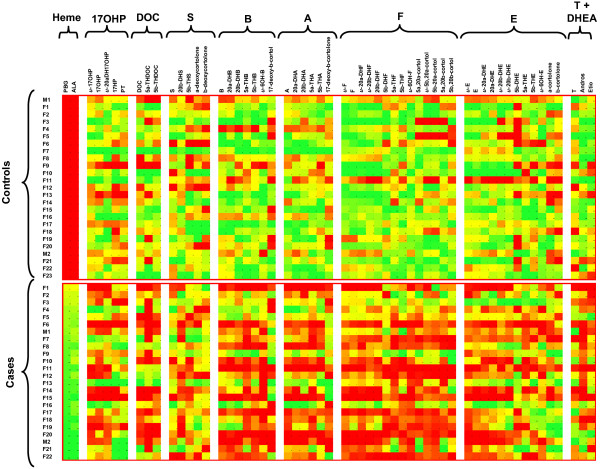
**Graphical by-patient representation of the corrected urinary concentrations for the analytes studied.** Green represents the 90% percentile, yellow the 50% percentile and red the 10% percentile. Lower concentrations (yellow-red tonalities) for cortisol and metabolites were found in AIP patients than in the control group (green-yellow tonalities). These differences were less significant in concentrations of cortisol precursors (17OHP, DOC and S) and their metabolites.

A graphic representation including all the patients (Figure 3) was constructed by calculating the percentile of every sample for each analyte in relation to the combined population group (cases + controls). A gradual color scale plot was performed using green for the highest values (90% percentile or higher), yellow for intermediate values (50% percentile) and red for the lowest values (10% percentile or lower).

## Results

### Analytes included in the metabolomic study

Forty-eight analytes fulfilled the criteria for acceptance (see “Statistical analysis” section) and were therefore included in the metabolomic analysis. These compounds are summarized in Figure 2. Most were predominantly excreted (>90%) as conjugated with glucuronide whereas 5 metabolites (6βOH-B, 6βOH-F, 6βOH-E, 20α-DH17OHP and 20α-DHF) were detected mainly as unconjugated. Seven compounds (17OHP, F, E, 20βDHF, 20βDHE, 20αDHE and β,α-cortol) were detected (>10%) in both forms and therefore the concentrations obtained in the unconjugated and total (unconjugated + conjugated) fractions were considered separately for the evaluation. In summary, 55 analytes were considered for the metabolomic study of steroid profiling in AIP patients.

### Case versus control groups

Case samples had heme precursor concentrations ranging from 3 to 64 nmol/mmol creatinine for PBG and from 1 to 40 nmol/mmol creatinine for ALA (normal values for PBG; <0.8; ALA < 5).

The steroid concentrations are summarized in Table 2. The urinary concentrations of the steroid were within the normal range in the control samples [18,20-22]. The concentrations found in the urine from AIP patients were generally lower for most of the analytes detected with significant differences (p < 0.05) in 41 out of the 55 steroids evaluated. Remarkably, differences increased with the number of hydroxylation processes undergone by the hormone. Thus, no significant differences (p > 0.05) were found in several metabolites of 17-hydroxyprogesterone, deoxycorticosterone and 11-deoxycortisol whereas 12 out of the 13 analytes related to cortisol (the most hydroxylated hormone) showed p values below 0.01 (the only exception was β, β-cortol with a p = 0.021). Generally, the p values for the 5β reduced metabolites were higher than those for their 5α counterparts. Decreased excretion of 5α-steroids compared to their 5β-counterparts has been previously described [12,23-26].

**Table 2:** Results found in control and AIP patients for the hormones and metabolites determined

	**T + DHEA**		**17OHP**		**DOC**		**S**	
	**Control**	**AIP patients**	**Control**	**AIP patients**	**Control**	**AIP patients**	**Control**	**AIP patients**
Parent	7.2	7.0	1.12	0.68	0.82	0.50	0.57	0.51
	(5.5-16.4)	(5.3-10.3)	(1.09-1.69)	(0.65-1.16)**	(0.65-1.27)	(0.41-1.05)	(0.45-0.81)	(0.38-0.66)
*u-*Parent	*n.d.*	*n.d.*	0.77	0.58	*n.d.*	*n.d.*	*n.d.*	*n.d.*
			(0.75-1.09)	(0.48-0.90)*				
20β-red.	*n.d.*	*n.d.*	*n.d.*	*n.d.*	*n.d.*	*n.d.*	1.19	0.61
							(1.06-1.49)	(0.56-1.0)**
*u-*20α-red.	*n.d.*	*n.d.*	0.45	0.28	*n.d.*	*n.d.*	*n.d.*	*n.d.*
			(0.39-0.57)	(0.20-0.42)**				
5β-red. + 3α-red.	2924	1993	200	240	15.1	6.5	41.8	36.3
	(2520–3636)	(1612–2642)**	(178–382)	(218–421)	(10.8-31.7)	(5.8-15.1)*	(40.8-71.4)	(33.8-50.2)
5α-red. + 3α-red.	3276	1262	*n.d.*	*n.d.*	2.4	0.1	*n.d.*	*n.d.*
	(2756–3820)	(1135–1990)**			(2.7-7.6)	(0.22-1.27)**		
5β-red. + 3α-red. + 20β-red.	*n.d.*	*n.d.*	983	1070	*n.d.*	*n.d.*	4.9	5.6
			(905–1346)	(883–1493)			(4.7-11.7)	(3.9-8.4)
5β-red. + 3α-red. + 20α-red.	*n.d.*	*n.d.*	*n.d.*	*n.d.*	*n.d.*	*n.d.*	8.5	7.7
							(7.0-12.5)	(6.6-10.5)
	**B**		**A**		**F**		**E**	
	**Control**	**AIP patients**	**Control**	**AIP patients**	**Control**	**AIP patients**	**Control**	**AIP patients**
Parent	7.8	3.1	48.5	22.2	119	38.4	227	135
	(7.0-14.6)	(2.7-6.9)**	(43.5-63.3)	(19.1-40.1)**	(91–153)	(32.0-52.6)**	(211–290)	(110–173)**
*u-*Parent	*n.d.*	*n.d.*	*n.d.*	*n.d.*	50.7	10.3	106	43
					(42.2-86.5)	(7.4-13.0)**	(99–175)	(39–69)**
*u-*6β-hydroxyl.	3.7	0	*n.d.*	*n.d.*	291	87	17.2	10.7
	(3.1-6.0)	(0.2-0.6)**			(265–408)	(77–122)**	(14.3-21.4)	(9.1-13.9)**
20β-red.	6.8	3.2	7.7	4.7	103	45	26.1	18.5
	(5.2-7.7)	(2.5-4.6)**	(7.1-9.9)	(3.4-6.9)**	(88–136)	(37–64)**	(23.4-31.7)	(17.0-25.7)*
*u-*20β-red.	*n.d.*	*n.d.*	*n.d.*	*n.d.*	62.3	24.3	13.2	7.2
					(55.5-99.3)	(18.1-33.0)**	(10.0-17.9)	(6.0-10.4)**
20α-red.	5.4	3.3	17.0	8.8	*n.d.*	*n.d.*	64.0	39.3
							(56.6-78.4)	(30.5-53.6)**
	(5.1-10.3)	(2.8-5.8)*	(14.5-20.7)	(6.7-15.7)**				
*u-*20α-red.	*n.d.*	*n.d.*	*n.d.*	*n.d.*	230	36	38.1	17.8
					(198–363)	(36–85)**	(34.2-52.6)	(14.3-27.0)**
5β-red.	*n.d.*	*n.d.*	*n.d.*	*n.d.*	6.9	4.3	9.4	13.0
					(5.6-8.1)	(3.2-4.9)**	(6.3-16.4)	(8.2-19.0)
5β-red. + 3α-red.	190	87	29.6	16.9	2009	1115	3665	2876
	(172–271)	(69–141)**	(24.7-40.5)	(14.0-31.6)*	(1751–2404)	(1040–1672)**	(3059–4325)	(2552–3743)
5α-red. + 3α-red.	308	89	8.1	2.8	967	350	56.6	31.8
	(308–470)	(89–211)**	(7.5-12.2)	(2.4-5.0)**	(912–1352)	(344–884)**	(53.6-89.7)	(25.9-61.7)*
5β-red. + 3α-red. + 20β-red.	16.2	9.1	61.8	31.6	111	81	476	575
	(9.9-25.0)	(7.2-20.0)	(50.7-91.7)	(32.7-94.8)	(94–158)	(70–115)*	(401–597)	(512–798)
5α-red. + 3α-red. + 20β-red.	*n.d.*	*n.d.*	*n.d.*	*n.d.*	35.4	14.0	*n.d.*	*n.d.*
					(26.4-41.3)	(13.1-26.7)**		
5β-red. + 3α-red. + 20α-red.	*n.d.*	*n.d.*	*n.d.*	*n.d.*	40.4	16.9	979	967
					(29.8-50.9)	(14.8-31.8)**	(830–1207)	(827–1264)
5α-red. + 3α-red. + 20α-red.	*n.d.*	*n.d.*	*n.d.*	*n.d.*	59.8	22.3	*n.d.*	*n.d.*
					(47.3-77.9)	(22.0-51.3)**		

AIP patients exhibited a significant decrease for all cortisol metabolites whereas concentrations in the normal range were found for most the metabolites from cortisol precursors. Results are expressed as median (μg/g creatinine) and 95% confidence interval for mean (in brackets) *P < 0.05, **P < 0.01 vs. control, “*u-*“ metabolite excreted unconjugated.

In order to check the individual status of every patient, Figure 3 provides a graphic representation of their steroid concentrations compared with the control group. For improving the clarity of the results, the patients were ordered by urinary PBG concentrations. In general, patients with relatively low heme precursor concentrations had urinary steroid concentrations closer to those of the control group, mainly showing yellow-orange tonalities in Figure 3. In contrast, AIP patients with higher concentrations of heme precursors had lower concentrations of steroids, showing predominantly orange-red tonalities in Figure 3. This decrease was more pronounced for cortisol, cortisone, corticosterone and 11-dehydrocorticosterone than for their 11-deoxycortisol, deoxycorticosterone and 17-hydorxyprogesterone precursors.

### Correlation of steroids versus heme precursors

**Figure 4:**
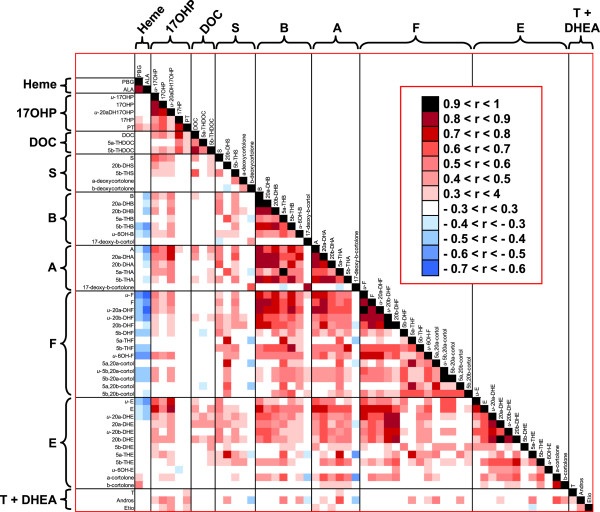
**Crossed correlation found in AIP patients between the 55 urinary steroids, PBG and ALA.** The expected positive correlation between hormones and their metabolites was obtained. Additionally, negative correlations between cortisol metabolites and heme precursors and positive correlations between 17-hydroxyprogesterone and heme precursors were found.

**Figure 5:**
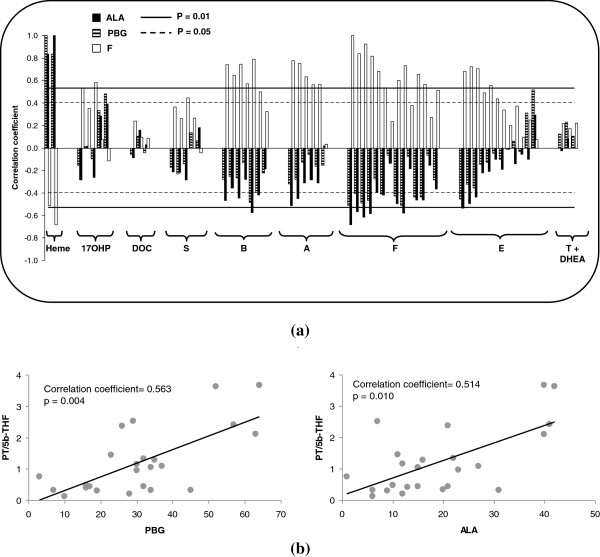
**Correlations between heme precursors and the analytes selected. (a)** correlation coefficients of PBG, ALA and F with all the analytes studied. Dotted and solid lines indicate a significance level at p = 0.05 and p = 0.01, respectively. The negative correlation between cortisol and heme precursors was of a similar magnitude to that of positive correlation obtained between cortisol and its direct precursor (corticosterone, B) or its direct product (cortisone, E). **(b)** correlation found between both heme precursors and the PT/5β-THF ratio (main metabolites of 17-hydroxy-progesterone and cortisol, respectively). The adrenal hormonal imbalance (ratio between the 17-hydroxy-progesterone precursor and the cortisol product) is correlated with the heme precursors detected in urine.

Based on the study by Christakoudi et al. in which steroid profiling was used for the study of 21-hydroxylase deficiency [27], a heat map was constructed showing the crossed correlations among the 55 urinary steroids, urinary PBG and ALA, found in AIP patients (Figure 4).This Figure, depicts the correlations found among all the analytes detected. Figure 5 provides the correlations found in AIP patients between heme precursors and the urinary steroids evaluated in greater detail. The correlation between cortisol and the remaining steroids is also included in Figure 5 as a model compound for steroid behaviour.

A positive correlation was found between cortisol and most of the urinary steroids, with this correlation being significant for most of the corticosterone, 11-dehydrocorticosterone, cortisol and cortisone metabolites evaluated (correlation coefficient between 0.4 and 0.9, p < 0.05). The expected positive correlation between PBG and ALA was also observed (correlation coefficient 0.86, p < 0.001).

Both heme precursors presented a negative correlation with most of the urinary steroids evaluated. The significance of this negative correlation increased with the number of hydroxylation steps performed in the biosynthesis of the hormone (see Figure 1), reaching a maximum for cortisol. Thus, 9 out of 14 cortisol metabolites showed a significant negative correlation with both PBG and ALA (correlation coefficient between 0.4 and 0.65, p < 0.05). The lowest correlation between heme precursors and cortisol metabolites was found for 5α-reduced metabolites. In general, the lowest significance in the correlation was found for polyreduced metabolites.

A positive significant correlation was observed between PBG and pregnantriol; i.e. the main metabolite of 17-hydroxyprogesterone (correlation coefficient = 0.482, p = 0.02). This positive trend was also found for the second main 17-hydroxyprogesterone metabolite (17HP), although statistical significance was not achieved (correlation coefficient = 0.334, p = 0.1).

Since an opposite trend was found between the urinary metabolites of the first product of the metabolic cascade (17-hydroxyprogesterone) and those of the end product (cortisol), several ratios were established between 17-hydroxyprogesterone and the cortisol metabolites in order to check the global function of steroidogenesis. Since hepatic 5α-reduction is decreased in AIP patients [12,23-26] and no 5α-metabolites are included in 17-hydroxyprogesterone, the use of 5α-reduced metabolites was avoided and only 5β-reduced metabolites were considered for the formation of ratios. The ratio between the main metabolites of 17-hydroxyprogesterone and cortisol (PT/5β-THF) showed a positive correlation with both PBG and ALA (correlation coefficient > 0.51, p < 0.01, see Figure 5b). Similar results were obtained for the remaining ratios tested.

## Discussion

The results presented in this study can be summarized into three main findings (i) urinary corticosteroid levels are decreased in AIP patients, (ii) cortisol and its metabolites are more decreased than its precursors in AIP, and (iii) the urinary concentration of cortisol metabolites is negatively correlated with the urinary concentration of heme precursors whereas an opposite trend may be observed for the main metabolites of 17-hydroxyprogesterone.

The study was performed with spot urine samples (second void of the morning) from AIP patient collected between 09.00 h and 10.00 h in a controlled hospital setting and not with at-home collected 24-hour urine. The possible weaknesses of a study based on spot-urine are minimized by the analysis of a large number of metabolites of each hormone. Since all cortisol metabolites were found to have a similar trend to a decrease in AIP patients (Table 2), our results seem to show a high degree of consistency, despite the use of spot morning urine.

The quantitation of a large number of hormones and metabolites also increased the significance of our findings. The measurement of a single metabolite (or a few metabolites) for each hormone would make the interpretation uncertain, since variations in urinary concentrations may be the consequence of either changes in the synthesis of a parent compound or changes in its metabolism (or a combination of both effects). In our study, every step of steroidogenesis was evaluated by different analytes, thereby increasing the reliability of the results as in the case of the decrease of cortisol which was supported by the decrease of 13 urinary species.

The overall decrease of corticosteroid excretion reported here is in agreement with the observations of Larion et al. [13] who found a decrease of cortisol in plasma in 3 AIP women with active disease. Moreover, we found a negative correlation between heme precursor concentrations in urine and most of the cortisol metabolites. Two tentative explanations can be hypothesized for our whole set of results: (i) AIP patients may present alterations in the hypothalamic pituitary adrenal (HPA) axis, and (ii) AIP patients may present deficiencies in the adrenal enzymes involved in the biotransformation of 17-hydroxyprogesterone to cortisol (Figure 1).

Neither of these hypotheses can be ruled out. However, if a decrease in pituitary-derived ACTH was the cause of the decrease in cortisol levels, a similar reduction would be expected for other adrenal corticosteroids. Our results showed normal values of 17-hydroxyprogesterone and 11-deoxycortisol metabolites suggesting that the adrenal activity was not reduced as a whole.

Light-sensitive extra-pituitary input from the suprachiasmatic nucleus regulated by the hippocampus induces a burst of cortisol secretion following morning awakening, the so-called cortisol awakening response (CAR) [28]. A possible dampening of CAR among AIP patients would also explain the decrease in cortisol observed in the morning urine. This effect would be more pronounced for those metabolites arising from rapid metabolism.

Acquired enzymatic deficiencies affecting the biosynthesis of cortisol could more likely explain the increased ratio between 17-hydroxyprogesterone metabolites (arising from the precursor) and cortisol metabolites (arising from the final product). This could be a consequence of heme deficiency associated with sustained up-regulation of ALAS-1 which is characteristic of active AIP. Heme deficiency could, in turn, partially decrease the activity of specific hemoproteins, notably P450 cytochromes, involved in steroid biosynthesis in the adrenal gland. Moreover, intracellular and mitochondrial energy deficiency, cofactor depletion or even direct ALA toxicity could also contribute to the changes observed in steroidogenesis.

It is unclear how the hormonal imbalance is associated with ALAS-1 induction and the sustained overproduction of heme precursors among AIP patients. It has been shown that steroids may induce ALAS-1 and porphyrin accumulation in liver cells [29]. Therefore, changes in adrenal metabolism could also be interpreted as physiological compensation mechanisms in situations of long-lasting ALAS-1 induction in the liver.

In conclusion, active AIP is associated with a hormonal imbalance of adrenal steroidogenesis; however, the effect of this imbalance on disease expression needs to be further evaluated.

## Abbreviations

AIP: Acute intermittent porphyria; HMBS: Hydroxymethylbilane synthase; ALAS-1: 5-aminolevulinate synthase; ALA: Aminolevulinic acid; PBG: Porphobilinogen; 17OHP: 17-hydroxyprogesterone; S: 11-deoxycortisol; DOC: Deoxycorticosterone; B: Corticosterone; A: 11-dehydrocorticosterone; F: Cortisol; E: Cortisone; T: Testosterone; DHEA: Dehydroepiandrosterone; DH: Dihydrometabolite; TH: Tetrahydrometabolite; 6βOH: 6β-hydroxy-metabolite; u-: Unconjugated metabolite; ISTD: Internal standard; HPA: Hypothalamic pituitary adrenal axis; ACTH: Adrenocorticotopic hormone; CAR: Cortisol awakening response.

## Competing interests

The authors declare that they have no competing interests.

## Authors’ contributions

OJP participated in the design of the study, carried out the steroidal analysis, participated in the discussion of the results and drafted the manuscript. JM helped in the conception of the study, in the steroidal analysis and in the discussion of the results. AF performed the statistical analysis. RV participated in the discussion of the results. PA recruited the patients. GC performed laboratory analyses, participated in the discussion of the results and participated in the study design. JS participated in the discussion of the results. JT participated in the design, coordinated the study, participated in the discussion of the results and wrote part of the manuscript. All authors read and approved the final manuscript.
